# The Symptoms Get Worse after Pregnancy in Sheehan's Syndrome: A Case Report

**DOI:** 10.1155/2012/271345

**Published:** 2012-09-19

**Authors:** Jingwen Hao, Min Liu, Zhaohui Mo

**Affiliations:** Department of Endocrinology, The Third Xiangya Hospital, Central South University, 138 Tongzipo Road, Changsha 410013, Hunan, China

## Abstract

Sheehan's syndrome, which is pituitary necrosis after severe postpartum hemorrhage and hypovolemia, may cause hypopituitarism immediately or several years later, depending on the degree of tissue destruction. We report an unusual case, in which a 55-year-old woman with Sheehan's syndrome got worse symptoms after spontaneous labor. In 1998, she had severe postpartum hemorrhage and pituitary necrosis during the third delivery, thus it was diagnosed as Sheehan's syndrome by clinical manifestations, laboratory tests, and magnetic resonance imaging. She was treated by replacement therapy including hydrocortisone and levothyroxine sodium. However, she had the fourth spontaneous pregnancy in 2000 and got worse symptoms after delivery. We carefully concluded that pregnancy provided no evidence against the diagnosis of Sheehan's syndrome because pregnancy might improve hypopituitarism by stimulating the pituitary remnant to undergo hyperplasia and irritating the placenta to secrete hormone. However, pregnancy might aggravate the symptoms by inducing autoimmunity as well. All in all, early diagnosis and adequate medical treatment are important to provide a good prognosis of Sheehan's syndrome.

## 1. Introduction

Sheehan's syndrome, which is pituitary necrosis after severe postpartum hemorrhage and hypovolemia, may cause hypopituitarism either immediately or after a delay of several years, depending on the degree of tissue destruction [1–3]. 40 years ago, it was estimated that the prevalence of Sheehan's syndrome was about 100–200 per 1,000,000 women [4]. In 2009, a retrospective nationwide investigation in Iceland reported that the prevalence of Sheehan's syndrome was estimated to be 5.1 per 100,000 women [5]. However, the pathogenesis of Sheehan's syndrome is still uncertain. Enlargement of pituitary gland, autoimmunity, small sella size, and disseminated intravascular coagulation (DIC) have been considered as important factors in the pathogenesis of Sheehan's syndrome. The clinical manifestation of Sheehan's syndrome varied from nonspecific symptoms like weakness, anemia, and fatigue to severe pituitary dysfunction resulting in coma and even death. Medical history of postpartum hemorrhage, failure to lactate, and cessation of menses are helpful clues to the diagnosis [6]. Early diagnosis and adequate medical treatment are crucial to reduce morbidity and mortality of the disease. 

The aim of this report is to describe a female patient who had the fourth spontaneous pregnancy after the appearance of postpartum hemorrhage and pituitary necrosis of the third delivery, and to critically focus on the symptoms getting worse after the fourth delivery in Sheehan's syndrome. This case report was conducted in accordance with the appropriate clinical and experimental ethical guidelines and was approved by the Ethical Committee of the Third Xiangya Hospital, Central South University. The case was reported with informed consent from the patient and her relatives. 

## 2. Case Description

The patient, a 55-year-old woman, was admitted to our hospital on February 3rd, 2012. Her chief complaint was repeated consciousness obstacle over two years. She had been in good health with normal menstrual cycles until her third delivery in 1988. She started to get mild hypofunction of gonad, thyroid, and adrenal cortex after severe postpartum hemorrhage. Unfortunately, owing to the limited medical resources in her hometown, she was not diagnosed as Sheehan's syndrome and did not receive enough professional treatment. Her menstrual periods prolonged (about 40–50 days) with little volume and short duration. In 1990, she conceived naturally for the fourth time but the symptoms got worse after delivery, especially in recent years. From 2000, she had unconsciousness for three times. She gradually had serious hypoglycemia, progressive fatigue, loss of cutaneous pigment, dryness, and furfuration of the skin, loss of muscle strength, and a decrease in libido after the fourth delivery. Nevertheless, she had no symptoms of vomiting, nausea, abdominal pain, or orthostatic dizziness. The family history of similar symptoms and previous history of hypertension or diabetes were not found.

**Figure 1:**
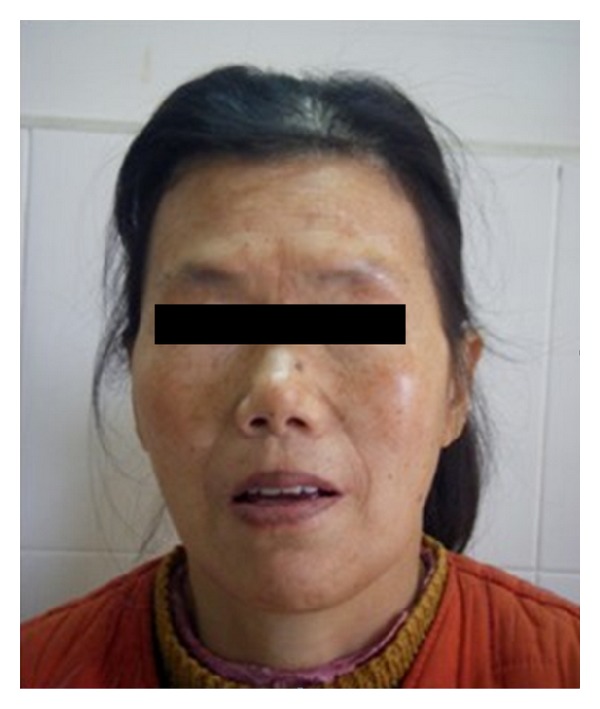
The eyebrows fall off.

The general physical examination showed that the blood pressure, heart rate, and temperature were within normal limits. She was in a coma and appeared chronically ill and pale with dryness and furfuration of the skin. Her eyebrows (Figure 1), glandebalaes and pubes fell off. There was no remarkable abnormality in lung, heart, and neurological examinations.

Routine laboratory findings demonstrated a red blood cell count of 3.32 × 10^12^/L, a hemoglobin of 101 g/L, a hematocrit of 30.3%, the random blood glucose 2.89 mmol/L, the serum sodium 121.0 mmol/L, the chloride 94.5 mmol/L, carbon dioxide combining power (CO_2_CP) 17.2 mmol/L, the uric acid 63 *μ*mol/L, the aspartate transaminase (AST) 72 U/L, the total protein (TP) 51.3 g/L, the albumin (ALB) 28.6 g/L, the globulin (GLB) 22.7 g/L, the triglyceride (TG) 2.83 mmol/L, the total cholesterol 7.62 mmol/L, the low-density lipoprotein cholesterol (LDL-C) 6.43 mmol/L, and the high-density lipoprotein cholesterol (HDL-CH) 0.67 mmol/L. The urine routine and stool routine were normal. 

As the clinical manifestations suggested Sheehan's syndrome, related endocrinological tests were finished as follows. The thyroid function test revealed a serum triiodothyronine (T3) of 0.31 ng/mL (normal 0.6 to 1.81), a thyroxine (T4) of 0 *μ*g/dL (normal 4.5 to 12.5), a free triiodothyronine (FT3) of 0.88 pg/mL (normal 1.8 to 4.2), a free thyroxine (FT4) of 0.16 ng/mL (normal 0.8 to 1.76), a thyroid-stimulating hormone (TSH) level of 1.547 *μ*IU/mL (normal 0.35 to 5.5), and a thyroglobulin autoantibody of 83.6 U/mL (normal 0 to 60). The plasma adrenocorticotropic hormone (ACTH) level was 4.8 pg/mL (normal 0 to 46). The plasma crotisol level was 0.75 *μ*g/dL (normal 3.9 to 22.4). The growth hormone (GH) level was 0.049 ng/mL (normal 0 to 10). The follicle-stimulating hormone (FSH) was 4.30 mIU/mL, the luteinizing hormone (LH) was 1.78 mIU/mL and the estradiol (E2) was 10.52 pg/mL. 

**Figure 2:**
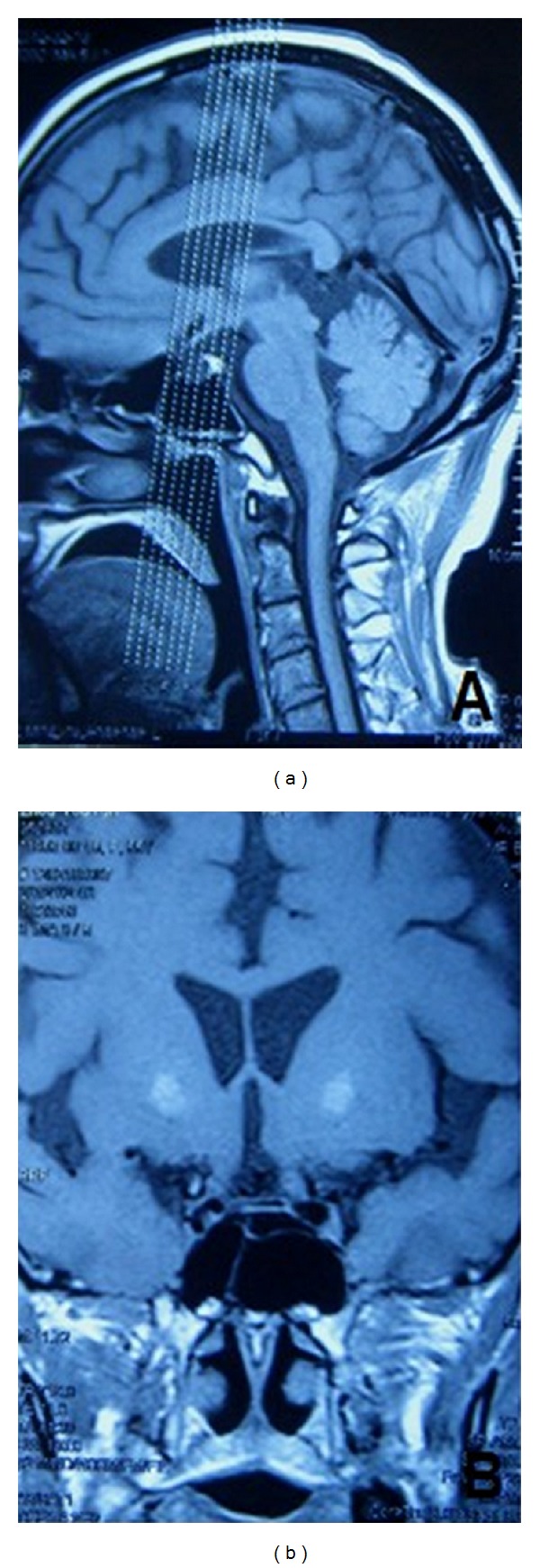
The sagittal (a) and coronary (b) noncontrast T1 weighted magnetic imaging (MRI) of the brain shows a diminutive pituitary and an empty sella turcica.

The brain magnetic resonance imaging (MRI) showed a diminutive pituitary and an empty sella turcica (Figure 2). There was no evidence of hemorrhage, intracranial mass, or aneurysm (Figure 2). 

She was diagnosed as Sheehan's syndrome and pituitary crisis. Replacement therapy was instituted with hydrocortisone and levothyroxine sodium. 

## 3. Discussion

Sheehan's syndrome occurs as a result of ischemic pituitary necrosis due to severe postpartum hemorrhage [7–10]. Only a small proportion of patients with Sheehan's syndrome may have a sudden onset of severe hypopituitarism immediately after delivery, whereas most patients have mild illness and they have not been diagnosed for a long time so that they are not treated appropriately [4]. Gei-Guardia et al. reported the average time between the previous obstetric event and diagnosis of Sheehan's syndrome was 13 years in a study of 60 patients [11]. In this case, although hypofunction of gonad, thyroid, and adrenal cortex had persisted, she had not been diagnosed until about 24 years later. At least 75% of pituitary have to be destroyed before clinical manifestations become evident [6]. Early diagnosis and treatment are crucial for the patients with Sheehan's syndrome especially for the patients with severe postpartum hemorrhage. In other words, we should pay attention to those patients who have postpartum hemorrhage.

This is a special and infrequent case with two features. First, she had the fourth spontaneous pregnancy after the appearance of postpartum hemorrhage and pituitary necrosis of the third delivery. Second, the symptoms of Sheehan's syndrome got worse after the fourth spontaneous pregnancy. 

Pregnancy occurring in the patients with Sheehan's syndrome is seldom described [12]. It is well known that the hypofunction of gonad and the effect of sex hormone axis are common in patients with Sheehan's syndrome. The cessation or disorder of menses would appear. So it is hard for pregnancy in Sheehan's syndrome. However, only a small proportion of patients with Sheehan's syndrome may have spontaneous pregnancy, which depends on the preservation of LH and FSH secretion after the pituitary apoplexy event. In this case, the patient could have the fourth spontaneous pregnancy after postpartum hemorrhage and pituitary necrosis because of the preservation of LH and FSH secretion. Grimes and Brooks also thought pregnancy does not constitute evidence against the diagnosis of Sheehan's syndrome [13].

Sheehan has reported that pregnancy might improve hypopituitarism by stimulating the pituitary remnant to undergo hyperplasia in Sheehan's syndrome [14]. As we all know, the pituitary gland is physiologically enlarged during pregnancy as a result of diffuse and nodular hyperplasia of the lactotroph cells stimulated by estrogens produced by the placenta [4]. The pituitary remnant may have an increment of 30 to 100 percent in the weight in patients with Sheehan's syndrome. It is also potential that placental secretion of TSH, estrogen, progesterone, and ACTH is responsible for the enhanced wellbeing of patients with hypopituitarism in Sheehan's syndrome. So pregnancy is propitious to the patients with Sheehan's syndrome, but it could improve the symptoms as well. See et al. [15] reported that spontaneous pregnancy could bring partial recovery of pituitary function in the patient with Sheehan's syndrome. However, in this case, the symptoms of Sheehan's syndrome got worse after the fourth spontaneous pregnancy. There are two possible reasons for this case. The first one is the long course of disease and the old age. The second one is the potential autoimmunity such as thyroglobulin autoantibody (83.6 U/mL in this case). Antipituitary antibodies are also speculated as the other autoimmune antibodies but without detection result because of the limited technical condition.

We report an unusual case with worse symptoms after spontaneous pregnancy in Sheehan's syndrome. In conclusion, pregnancy provides no evidence against the diagnosis of Sheehan's syndrome because pregnancy might improve hypopituitarism by stimulating the pituitary remnant to undergo hyperplasia and stimulating placenta to secrete hormone, however, pregnancy might also aggravate symptoms by stimulating autoimmunity system. Early diagnosis and appropriate treatment are necessary and important.

## References

[1] Laway BA, Mir SA, Bashir MI, Bhat JR, Samoon J, Zargar AH (2011). Prevalence of hematological abnormalities in patients with Sheehan’s syndrome: response to replacement of glucocorticoids and thyroxine. *Pituitary*.

[2] Gokalp D, Tuzcu A, Bahceci M (2011). Analysis of thrombophilic genetic mutations in patients with Sheehan’s syndrome: is thrombophilia responsible for the pathogenesis of Sheehan’s syndrome?. *Pituitary*.

[3] Sert M, Tetiker T, Kirim S, Kocak M (2003). Clinical report of 28 patients with Sheehan’s syndrome. *Endocrine Journal*.

[4] Keleştimur F (2003). Sheehan’s syndrome. *Pituitary*.

[5] Kristjansdottir HL, Bodvarsdottir SP, Sigurjonsdottir HA (2011). Sheehan’s syndrome in modern times: a nationwide retrospective study in Iceland. *European Journal of Endocrinology*.

[6] Shivaprasad C (2011). Sheehan's syndrome: newer advances. *Indian Journal of Endocrinology and Metabolism*.

[7] Lee YS, Moon SS (2011). A case of sheehan’s syndrome that manifested as bilateral ptosis. *Journal of Korean Medical Science*.

[8] Tessnow AH, Wilson JD (2010). The changing face of Sheehan’s syndrome. *American Journal of the Medical Sciences*.

[9] Kaplun J, Fratila C, Ferenczi A (2008). Sequential pituitary MR imaging in Sheehan syndrome: report of 2 cases. *American Journal of Neuroradiology*.

[10] Pasa S, Altintas A, Tumer C (2010). Prothrombin time, activated thromboplastin time, fibrinogen and D-Dimer levels and von-willebrand activity of patients with Sheehan’s syndrome and the effect of hormone replacement therapy on these factors. *Uluslararasi Hematoloji-Onkoloji Dergisi*.

[11] Gei-Guardia O, Soto-Herrera E, Gei-Brealey A, Chih Hao CK (2011). Sheehan syndrome in Costa Rica: clinical experience with 60 cases. *Endocrine Practice*.

[12] Vieira HB, Knoepfelmacher M, Salgado LR, Wajchenberg BL, Liberman B (1995). Preservation of gonadotrophic function and pregnancy in Sheehan’s syndrome: a case report and review of the literature. *Revista da Associação Médica Brasileira*.

[13] Grimes HG, Brooks MH (1980). Pregnancy in Sheehan’s syndrome. Report of a case and review. *Obstetrical and Gynecological Survey*.

[14] Sheehan HL, Murdoch R (1938). Postpartum necrosis of the anterior pituitary. Effect of subsequent pregnancy. *The Lancet*.

[15] See TT, Lee SP, Chen HF (2005). Spontaneous pregnancy and partial recovery of pituitary function in a patient with Sheehan’s syndrome. *Journal of the Chinese Medical Association*.

